# The *Saccharomyces* Genome Database—a history of ideas and accomplishments, 1994–2026

**DOI:** 10.1093/femsyr/foag042

**Published:** 2026-08-25

**Authors:** J Michael Cherry, Gavin Sherlock, Stacia R Engel

**Affiliations:** Department of Genetics, Stanford University, Stanford, CA 94305, USA; Department of Genetics, Stanford University, Stanford, CA 94305, USA; Department of Genetics, Stanford University, Stanford, CA 94305, USA

**Keywords:** *Saccharomyces* Genome Database, eukaryotic biology, history, genetics, functional genomics

## Abstract

The *Saccharomyces* Genome Database (SGD) is one of the longest-running and most consequential biological databases in the world. Founded in the early 1990s at Stanford University under the visionary leadership of David Botstein and developed under the long-term technical direction of J. Michael Cherry, SGD has served for more than three decades not only as the authoritative knowledge center for the budding yeast *Saccharomyces cerevisiae*, but also as the source for much of the fundamentals of eukaryotic biology. This history traces the arc of a remarkable intellectual and scientific project: beginning with the challenge of building the very first integrated eukaryotic genome database and evolving across 30 years into a global knowledge hub for genetics, functional genomics, and human disease research. The history is organized chronologically, with each section highlighting the central ideas, technical developments, and concrete accomplishments of that period.

## The founding vision

### Context and rationale—we have a problem

In 1994, the Human Genome Project had been underway for several years; in addition to sequencing the Human Genome, it was also proposed as part of the project to sequence multiple model organisms, namely yeast, mouse, *Drosophila melanogaster*, and *Caenorhabditis elegans*. As data were generated, the scientific community was beginning to grapple with a problem that had no precedent: how to store, organize, retrieve, and make biologically meaningful a vast and rapidly growing body of genomic information. The challenge was stark: no satisfactory database system for integrated genomic information existed.

### The Rosetta Stone

The choice of *Saccharomyces cerevisiae* as one of the model organisms to be sequenced was deliberate and forward-looking. Budding yeast had the smallest eukaryotic genome then being sequenced (12.5 megabase pairs, 1/230th the size of the human genome), a vast existing body of genetic knowledge, and a remarkable degree of evolutionary conservation with the human genome. Roughly one-third of yeast genes were already known to have human counterparts. Examples such as the yeast *IRA1/IRA2* genes being orthologous to the human neurofibromatosis NF1 gene, and yeast *MSH2* revealing the mechanism of human colon cancer, made the case that a well-curated yeast genome database could serve as a “Rosetta Stone” for interpreting human gene sequences. This foundational argument, that yeast biology could unlock human biology, remains the intellectual backbone of the project. Interpreting the human genome would require well-annotated model organism genomes as functional references.

### Origins and collaborators—we have a solution

*Saccharomyces* Genome Database (SGD) arose from a collaboration between three scientists who had each independently built foundational resources for the yeast community. Robert Mortimer (UC Berkeley) had maintained the community’s genetic map for over 30 years, publishing updated versions every few years and accumulating 1898 mapped loci, making it the most complete genetic/physical map of any eukaryote at the time (e.g. Mortimer et al. 1992). Maynard Olson (University of Washington) had constructed a complete physical map (e.g. Riles et al. 1993) covering 99% of the yeast genome using cosmid and lambda clone ordering. David Botstein (Stanford) served as Principal Investigator and provided the institutional home and scientific vision. Both Mortimer and Olson were looking to pass on their accumulated data to the community without committing to years more of curation. SGD was the solution.

J. Michael Cherry served as technical director of the SGD group, and this arrangement—a biologist of Botstein’s stature providing scientific direction, paired with Cherry’s technical leadership and a team of PhD-level curators—would define the project for decades.

### The curator team: building expertise over decades

From the beginning, SGD was built on a model that was unusual for database projects: PhD-level scientists working as full-time curators. This was not a temporary position for postdocs *en route* to faculty careers, but a professional role requiring deep biological knowledge, critical reading skills, and long-term institutional memory.

### The importance of context

The founding curator team established a pattern that would define SGD for decades, and many curators remained with the project for 10, 15, even 20 years or more. This continuity proved essential: curators developed expertise not just in yeast biology broadly, but in specific areas. One might become the expert on ribosome biogenesis, another on cell cycle regulation, a third on metabolic pathways. When a new paper appeared, the curator who read it brought not just general knowledge but years of context about what was already known, what controversies existed, and how the new findings fit into the larger picture.

### Trust is built

Curators attended the major yeast meetings including the biennial Yeast Genetics and Molecular Biology Meeting, the Cold Spring Harbor Yeast Cell Biology meeting, and other conferences, not as passive observers but as active participants. They presented SGD updates, solicited feedback, answered questions at the SGD booth, and, most critically, talked with researchers about their work and about their data. These face-to-face interactions built trust and revealed what the community actually needed, often in ways that surveys and emails could not capture.

## Early accomplishments

### What a hit

By September 1994, the first full beta-test version of SGD was released online. At the Yeast Genetics and Molecular Biology Meeting in Seattle, the demonstration drew enthusiastic feedback. Researchers who had struggled to track down genetic maps from scattered publications, or who had spent hours manually searching GenBank for related sequences, immediately grasped the value of having integrated, cross-referenced information accessible from a single website. The response was not just approval but relief: the community had been waiting for exactly this resource. The database was accessible via Gopher, a pre-Web internet protocol, and the nascent World Wide Web through SacchDB, derived from ACeDB (Eeckman and Durbin 1995), and already integrated genetic maps (updated from Mortimer), physical maps (updated from Olson), DNA sequences from sequencing groups and GenBank, and biological information curated from the literature, all cross-referenced and hyperlinked. Usage was already meaningful: ~1000 different Internet computers per month were connecting to the Gopher server, matching the estimated number of yeast research laboratories in the world at the time. The long-term goal articulated in 1994 was ambitious: to bring SGD to a state where the entire yeast genome sequence was annotated and connected to all available biological information.

### Find a friend or meeting

SGD also inherited and integrated community infrastructure that predated the database itself. In 1985, Terrance Cooper began publishing a telephone directory of yeast researchers, which grew through several editions and was transferred to special supplemental editions of Yeast in 1992 and 1993 before being integrated into SGD’s “Community” section in 1994 (Dujon and Cooper 2025). This Colleagues database, with thousands of entries and built using ACeDB’s existing class structure, allowed researchers to find collaborators and identify experts in specific research areas.

From its earliest days, SGD became the central hub for community information about yeast research. The database served as a clearinghouse for meeting and conference announcements, ensuring researchers were informed about relevant scientific gatherings worldwide. For many years, SGD handled the organization and hosting of submitted abstracts for the International Conference of Yeast Genetics and Molecular Biology Meeting, one of the field’s premier conferences. SGD also archived meeting materials including abstracts, participant lists, and photographs from the Yeast Genetics Meeting, preserving the community’s history and making past scientific presentations accessible. These community-oriented services established SGD as not merely a data repository but as the organizational and social hub of yeast research.

## The genome is sequenced

### A transformed landscape—55 000 views per week

The yeast genome had been completely sequenced by April 1996 through a world-wide collaborative effort (Goffeau et al. 1996). *Saccharomyces cerevisiae* was the first eukaryote to have its entire genome sequenced, which fundamentally changed the nature of SGD’s mission (Botstein and Cherry 1997, Botstein et al. 1997)). The challenge of mapping was over; the challenge of annotation and biological interpretation had arrived in full.

**Figure 1:**
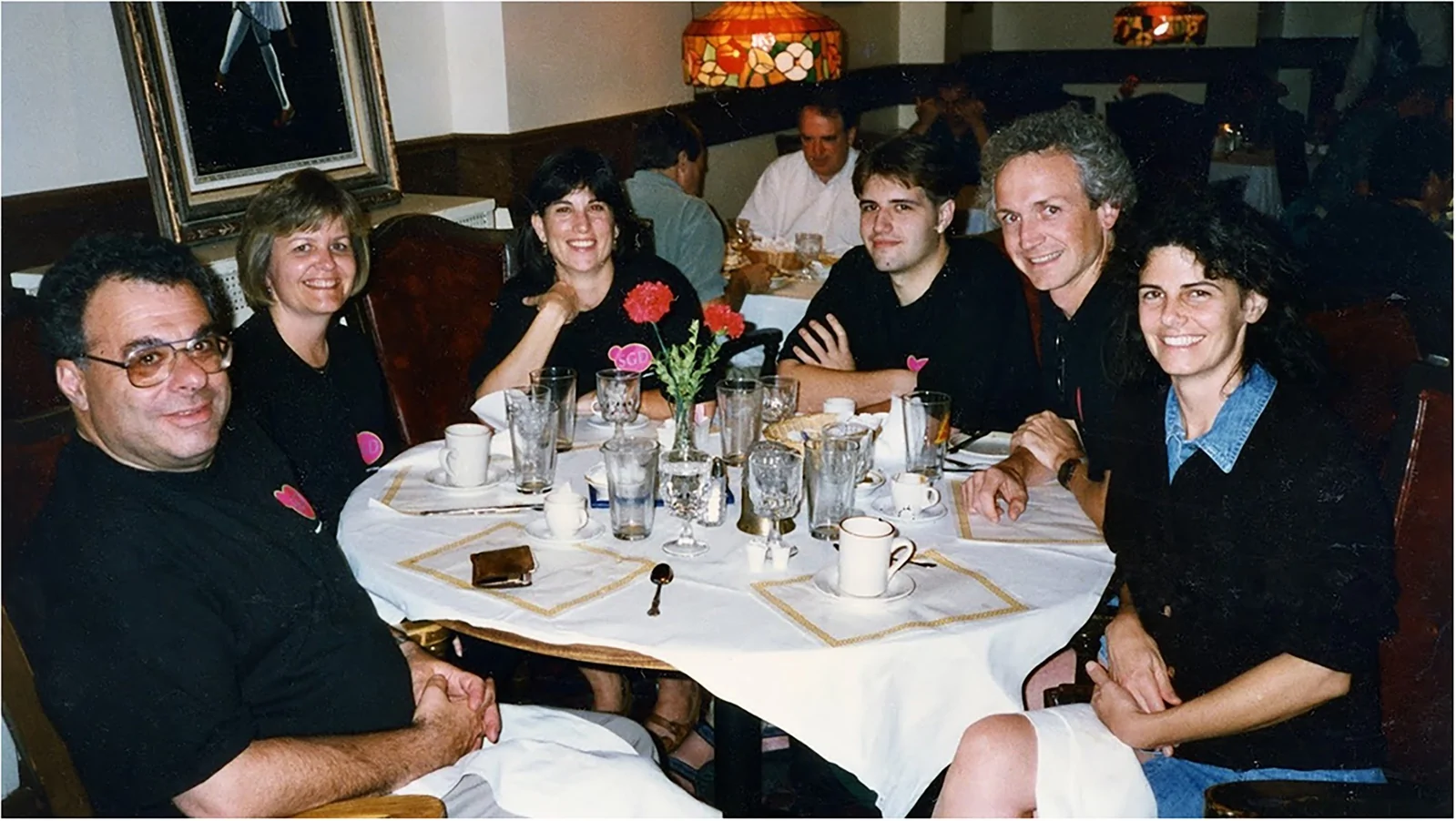
The SGD group at the 1996 Yeast Genetics and Molecular Biology Meeting in Madison, Wisconsin. From left to right, David Botstein, Gail Binkley, Cathy Ball, Paul Spellman, Mike Cherry, and Barbara Dunn.

The completed genome presented a challenge that became one of SGD’s most enduring intellectual contributions: the idea that the yeast genome could serve as a “rigorous foundation for annotation of a major subset of all eukaryotic proteins.” As collaborator Temple Smith (Boston University) argued, annotation errors propagate (Smith and Zhang 1997)—poor information in one database corrupts others—making a high-quality, carefully maintained reference genome essential for the entire field. By 1997, SGD received more than 55 000 web page views per week (Fig. 1).

### Major new features

The period between 1994 and 1997 saw a rich expansion of SGD’s content and tools. Sacch3D provided BLAST reports integrating 3D structural information for yeast proteins (Cherry et al. 1998). Using BLASTP analysis against the Brookhaven Protein Data Bank, 934 yeast ORFs (15% of the genome) were linked to known structural data, with visualization of homology regions in interactive 3D viewers and connections to the SCOP domain classification system. Gene_Info provided a curated literature guide system linking abstracts to biological topics for each gene. The new graphical interface was a major leap from previous text-based access to information. The Combined Physical and Genetic Map, the Genome View (with Java-based chromosome browsing), the Chromosomal Features Map, and “minimap” snippets all appeared during this period (Cherry et al. 1997). Sequence tools such as the Global Gene Hunter (simultaneous cross-database search), Design Primers (PCR primer design from genomic sequence), Sequence Similarity Viewer (dot-matrix genome self-comparison), and an early virtual Southern/restriction analysis tool allowed researchers to explore the emerging genomic data. Downloadable information such as Chromosomal Features Table and FTP data files allowed bulk tabular data downloads enabling computational analysis of the entire genome.

### Conflicts resolved

Perhaps most significantly, with the Gene Registry, SGD formalized its role as the authority for yeast gene nomenclature, taking over from Robert Mortimer who had maintained this responsibility for the community for decades. The transition preserved Mortimer’s careful stewardship while modernizing the process: researchers could submit gene names through a web-based form and receive confirmation within 1–2 business days. This seemingly administrative function was actually a critical community service. A researcher who discovered a new gene and wanted to name it needed to know immediately whether their chosen name was already taken, whether it fit nomenclature conventions, and whether it might be confused with existing genes. SGD curators handled these requests personally, often suggesting alternatives when conflicts arose and maintaining a consistent naming system across thousands of genes. By 1997, over 300 gene names were being registered annually, each one representing a new discovery and a direct interaction between a researcher and SGD.

### Biochemical pathway curation and community collaborations

In collaboration with Doug Brutlag’s lab at Stanford, a motif-based approach assigned functional classes to then-uncharacterized yeast proteins, with results cross-validated against Sacch3D structural predictions (Nevill-Manning et al. 1998). These computational predictions were subsequently refined through experimental validation as the yeast community systematically characterized these genes. Collaborations with Dan Voytas (Iowa State) enabled systematic identification and naming of all yeast Ty transposable elements, LTRs, and tRNAs (Kim et al. 1998). SGD also assumed responsibility for maintaining and updating the systematic sequence for chromosomes sequenced by the Stanford DNA Sequence Center directed by Ron Davis, Washington University directed by Mark Johnston, and McGill University directed by Howard Bussey.

## Gene Ontology and scalable database infrastructure

### Usage at scale and community impact

By 2000, SGD was receiving over 80 000 page views per week, with 6000 unique hosts connecting monthly. More than 13 million cumulative hits had been logged. The database had become deeply embedded in the practice of yeast research, with many researchers using it daily to access integrated genomic information, evaluate gene annotations, and explore functional relationships.

### The birth of Gene Ontology—built on trust

Perhaps the most consequential initiative launched in this period was the Gene Ontology (GO) project. The collaboration began with conversations between Michael Ashburner (FlyBase), Judith Blake (Mouse Genome Informatics), and Mike Cherry (SGD) at meetings and via email in the late 1990s. Each database faced the same problem: their annotations used different vocabularies, making cross-organism comparisons nearly impossible (Gene Ontology Consortium 2001). The solution required not just technical work but trust: three databases serving different communities agreeing to build something together that none could own exclusively. SGD joined with FlyBase and Mouse Genome Informatics to develop three independent but interoperable ontologies: Biological Process (why), Molecular Function (how), and Cellular Component (where). This collaboration addressed a fundamental problem in postgenome biology: without a shared, controlled vocabulary, biological annotations from different organisms and databases could not be meaningfully compared or integrated.

### It connected the organisms

The collaboration was genuinely joint; curators from all three databases participated in conference calls, debated term definitions, and tested the ontologies on their own organisms. By 2000, SGD curators had already associated GO terms with 1700 yeast genes. Published in Nature Genetics (Ashburner et al. 2000), GO would go on to become the standard vocabulary for functional annotation across virtually all organisms and databases in biology, and a testament to what model organism communities could achieve together. It has had an enormous impact on interpreting the ever-growing lists of genes that resulted from genomic studies because the structure of the GO offers several powerful features that other bioontologies have since adopted, including a system of terms—such as function or process names—that are human-readable yet designed for computational use. Many computational tools have been built over the years that leverage GO to map relationships and explore cellular pathways (e.g. Boyle et al. 2004). Using GO annotations together with the GO ontology structure, researchers can group genes to analyse many types of experimental results (Dwight et al. 2002).

### The oracle transition

A major internal milestone at SGD was the migration from ACeDB to a relational database management system (Oracle RDBMS). This shift from a specialized academic platform to industry-standard software enabled more sophisticated data querying, improved scalability, and the dynamic generation of web pages. These capabilities became essential as the database grew in complexity and usage, with exponential growth in the numbers of annotations being generated and stored. The transition was staged carefully through the year 2000, with graphical displays, colleague information, and genetic mapping data sequentially moved to the Oracle backend.

### Diminishing information overload

The introduction of the Gene Summary Paragraph represented a new form of expert synthesis in which curators moved beyond restating published abstracts and began to provide selective, interpretive summaries of what was known about a gene. This was an early recognition that information overload from the accumulation of thousands of annotations required human intelligence to distill into a coherent biological narrative.

## Comparative genomics and reference genome stewardship

### The postgenome era

By 2005, the Human Genome Project had published its working draft (2001) and finished sequence (2004). The genomes of *C. elegans* (*C. elegans* Sequencing Consortium 1998), *D. melanogaster* (Adams et al. 2000), and *Schizosaccharomyces pombe* (Wood et al. 2002) ) had also been sequenced. Using BLASTP analyses, SGD showed that 1856 yeast proteins matched fly proteins and 1704 matched worm proteins (Chervitz et al. 1998), demonstrating the utility of well-annotated model organism databases for understanding the entire eukaryotic kingdom. SGD received more than 200 000 visits per week by this point.

### Reference sequence curation and annotation

An enormous effort was required to maintain the S288C reference genome sequence, not simply as a static record, but as an evolving, curated document. Since 2000, SGD has investigated 161 proposed sequence changes using comparative genomics data, confirming 94 genuine corrections to the original 1996 sequence (Fisk et al. 2006, Engel et al. 2014). More broadly, the genome annotation had expanded with over 500 new ORFs, RNA genes, ARS elements, and telomeric features, and individual ORF start codons and intron/exon structures had been revised for scores of genes.

### How good are the data

A critical contribution of this period was the classification of ORFs into three categories—Verified, Uncharacterized, and Dubious—to address the long-standing debate about how many of the originally predicted ORFs were genuinely protein-coding. Rather than silently removing suspect ORFs, SGD adopted a transparent system in which Dubious ORFs were retained in the database but clearly labeled, promoting further experimentation (Christie et al. 2009). By 2005, 65% of all open reading frames had been experimentally characterized, a benchmark unprecedented for any eukaryote at that time.

### Curation pipeline and literature infrastructure

By 2005 the project was devoting significant effort to the science of curation itself. SGD had built a comprehensive pipeline for identifying relevant yeast literature, including automated PubMed queries that were refined to capture papers using “yeast” rather than only “cerevisiae,” and to handle electronic prepublications. The curators worked from a careful division: first identifying papers containing novel data, then reading them in full to capture sequence and annotation changes, GO annotations, phenotype data, and literature topics. Archiving sequence versions was formalized so that researchers using historical datasets could determine exactly which genome release corresponded to their data.

## Phenotype ontologies, high-throughput data, and infrastructure maturation

### Building the yeast phenotype ontology

SGD celebrated “the awesome power of yeast genetics,” the tradition of genetic analysis that had defined the field for decades, from classical mutant screens to the landmark systematic deletion collection covering the entire yeast genome. To properly represent this genetic wealth, SGD developed a new controlled vocabulary: the Yeast Phenotype Ontology (Costanzo et al. 2009), later renamed the Ascomycete Phenotype Ontology (APO; Engel et al. 2010) as it was adopted by the *Candida* and *Aspergillus* Genome Databases. The APO enabled curators to capture not just that a phenotype existed, but its direction (increased/decreased relative to wild type), the nature of the mutation, the experimental method, the strain background, and the growth conditions—a richly multidimensional annotation system.

### Ready for prime time

This was a major curatorial undertaking. SGD reviewed classical genetics literature going back to Hartwell’s foundational CDC gene studies (Hartwell et al. 1970, Hartwell 1971a, b, Culotti and Hartwell 1971), curated genome-wide deletion phenotype datasets, and conducted consistency exercises among curators to ensure uniform standards. The result was nearly 21 000 phenotype annotations from classical genetics studies and over 43 300 from large-scale experiments, covering more than 6000 chromosomal features. By summer 2008, when the new phenotype system was unveiled at the Yeast Genetics and Molecular Biology Meeting in Toronto, the response was enthusiastic.

### A new reference sequence

In February 2010, SGD released the first major update of the S288C reference sequence since 1996 (Engel et al. 2014). Generated by Fred Dietrich (Duke University) using Illumina sequencing of the AB972 strain (a direct S288C descendant), the update changed 194 protein sequences and was applied to all 16 chromosomes. From this point, any further differences between laboratory versions of ostensibly the same strains would be treated as sequence variation, i.e. alleles, rather than corrections, marking a conceptual maturation in how the reference genome was maintained.

### High-throughput data integration

The explosion of genome-wide experimental techniques such as transcription profiling, ChIP-chip, nucleosome positioning, promoter occupancy, and Single Nucleotide Polymorphism (SNP) mapping produced vast datasets that were scattered across publisher and laboratory websites in incompatible formats. A major development in 2011 was the addition of 17 high-throughput datasets into an upgraded GBrowse genome browser, allowing users to view diverse data types in genomic context (Cherry et al. 2012). This represented a qualitative shift: SGD was no longer only an annotation resource for individual genes but a platform for integrating and visualizing the outputs of modern genomics.

### Usage and community feedback—cannot live without it

Community feedback provided rich evidence of what researchers actually needed from SGD and how they used it. Yeast researchers universally considered SGD valuable, with most using it frequently, many daily. The written comments revealed something deeper. Researchers described SGD as “indispensable,” “the first place I go,” and “what I show to my students on day one.” Some noted that they kept an SGD window open continuously while working. Others described how SGD had changed their research practice: instead of reading papers linearly, they would look up genes first, see what was already known, and then read primary literature with that context in mind. One researcher noted simply: “I don’t know how we worked before SGD existed.”

### Unbelievable complexity

The Locus Summary page consistently accounted for the largest share of page views, confirming that the gene-centric view was the anchor of scientific use. Among curated data types, Mutant Phenotype, Protein and Genetic Interactions, and Summary Paragraphs were the most valued. Perhaps most telling was that a notable fraction of users did not work on *S. cerevisiae* at all, confirming that SGD had become indispensable beyond the yeast community. Visitors from countries around the world were recorded.

Infrastructure had grown correspondingly complex: SGD was managing 18 different database instances (Oracle, MySQL, and PostgreSQL), reflecting the diverse software ecosystems required to support specialized tools like genome browsers and data warehouses. This infrastructure was necessary for supporting applications including GBrowse (Stein et al. 2002) and an InterMine-based (Sullivan et al. 2013) data warehouse (YeastMine; Balakrishnan et al. 2012) that allowed complex cross-queries of all SGD data types.

## The richness of multidimensional annotation

### More than the sum of the pieces

As experimental technologies matured and the yeast research community generated increasingly diverse types of data, SGD evolved from a repository of genome sequence and basic gene function into a multidimensional molecular atlas. This transformation was driven not by SGD alone, but by a productive symbiosis between database curators and experimental researchers: the community generated rich datasets, and SGD developed the infrastructure to integrate, visualize, and make them accessible.

### Protein complexes and biochemical pathways

The shift from studying individual genes to understanding biological systems required new organizational frameworks. SGD systematically curated protein complex composition, integrating data from affinity purification–mass spectrometry studies, yeast two-hybrid screens, and targeted biochemical analyses (Wong et al. 2019). The result was a comprehensive map of how yeast proteins assemble into functional machines, providing information essential for interpreting genetic interactions and phenotypes.

In parallel, SGD developed YeastPathways, a comprehensive biochemical pathways resource built on the BioCyc platform. This resource moved beyond the question “what does this gene encode?” to “what cellular processes does this gene participate in?” By linking genes to metabolic reactions, regulatory circuits, and biosynthetic routes, YeastPathways provided the context necessary to understand how mutations in individual genes produce systems-level phenotypes.

### From sequence variation to functional consequence

The recognition that laboratory strains of *S. cerevisiae* differ substantially from one another, with wild isolates harboring even greater diversity, created both a challenge and an opportunity. In 2015, SGD introduced the Variant Viewer (Sheppard et al. 2016) an interactive web tool that displays sequence differences between the S288C reference genome and other commonly used laboratory strains and wild isolates. Critically, the tool visualizes variation at both the nucleotide and amino acid level, allowing researchers to immediately assess whether a sequence difference is likely to alter protein function.

## That strain does not fit my needs

This capability proved essential as the community increasingly used diverse genetic backgrounds for specialized studies: some strains are preferred for mitochondrial research, others for mating-type switching, still others for industrial fermentation studies. The Variant Viewer enabled researchers to determine whether a phenotypic difference between strains reflected the specific mutation under study or a background polymorphism, a question that had previously required laborious manual sequence comparison.

### Post-translational modifications: the dynamic proteome

Proteins are not static molecules; their activity, localization, and stability are dynamically regulated through chemical modifications. SGD’s curation of post-translational modifications (PTMs) captured this regulatory layer, accumulating 44 204 PTM annotations covering 3639 yeast genes from 310 publications (Hellerstedt et al. 2017) in the initial public release. Phosphorylation dominated with 35 161 annotations, followed by ubiquitination, succinylation, acetylation, and methylation, among others. Curation is ongoing: 154 775 PTM annotations covering 5041 yeast genes from 731 publications are available. This rich dataset enables researchers to investigate how protein activity is dynamically regulated in response to environmental signals, cell cycle progression, and metabolic state. The data are accessible through both the SGD website and YeastMine, allowing users to ask questions such as: *Which kinases phosphorylate this protein? Which sites are modified under stress? Which proteins are ubiquitinated during mitosis?*

### Quantitative dimensions: expression and abundance

Genome annotation traditionally focused on qualitative information such as gene structure, function, and localization, but biological understanding increasingly demanded quantitative data. *How much of each protein is present in a cell? How does expression change across growth conditions, developmental stages, or genetic perturbations?*

In 2020, SGD incorporated a unified protein abundance dataset (Nash et al. 2020), synthesizing measurements from multiple proteomics studies to provide consensus estimates of protein copy number per cell. This quantitative layer transformed how researchers could use SGD: instead of simply knowing that two proteins interact, one could now assess whether they are coexpressed at levels consistent with stoichiometric complex formation.

SGD also developed transcriptome visualization tools, integrating RNA sequencing datasets and microarray studies to display gene expression patterns across hundreds of experimental conditions (Ng et al. 2020). The Expression Atlas allowed users to ask: *Is this gene induced by heat shock? Repressed during stationary phase? Coregulated with other members of a pathway?* These questions, central to understanding gene function, required not just cataloging individual experiments but creating a queryable, integrated view across the entire body of expression data.

### Protein localization at genome scale

Knowing where a protein acts is often as informative as knowing what it does. SGD curated protein localization data from both small-scale studies and genome-wide fluorescence microscopy projects that systematically tagged and imaged thousands of yeast proteins (Huh et al. 2003). The integration of these datasets, ranging from high-resolution confocal images to high-throughput automated imaging, provided subcellular localization information for the majority of the yeast proteome.

### Generating hints

These localization data, represented in GO Cellular Component terms, enabled sophisticated queries: Which uncharacterized proteins localize to mitochondria? Which transcription factors concentrate in nuclear foci under stress? Which membrane proteins redistribute during the cell cycle? The answers to such questions often suggest functions even for genes with no other annotations.

### Community data contributions—more than papers

SGD’s richness depended fundamentally on data contributed by the research community. While curators extracted information from published papers, researchers also contributed directly by submitting unpublished data, correcting errors, updating gene names, and sharing preliminary results that helped contextualize published findings. Some contributions were systematic: the deletion collection project (Giaever and Nislow 2014), the GFP localization studies (Huh et al. 2003), the global two-hybrid screens (Ito et al. 2000, 2001, Uetz et al. 2000). These consortia generated data for thousands of genes simultaneously and deposited it with SGD for curation and distribution. Other contributions were individual: a researcher noticing that a gene annotation was outdated, or that two genes had been conflated, or that an intron boundary was incorrectly annotated. SGD formalized this community engagement over decades through the “Colleagues” system, which allowed researchers to create profiles which were linked to genes they studied. This created a network of distributed expertise: for nearly every well-studied gene, there were one or more researchers who knew that gene intimately and who could be contacted when questions arose. The relationship was reciprocal. Researchers contributed data and expertise; SGD provided infrastructure, quality control, and long-term stewardship. Neither could succeed without the other.

### Tools for training and discovery—a new core competency

As SGD’s data holdings expanded, so did the need to teach researchers how to use them. SGD launched a webinar series and developed extensive educational resources, providing interactive training sessions that reached researchers worldwide. These webinars covered both practical skills, such as how to search for genes with specific phenotypes or how to download datasets for computational analysis, and conceptual guidance on interpreting complex annotations.

The educational mission extended beyond research laboratories. SGD became a standard resource in undergraduate and graduate genetics courses, with instructors using the database to teach students how to navigate the primary literature, understand experimental evidence codes, and think critically about gene function prediction. This formalization of SGD’s educational role represented a recognition that database literacy had become a core competency in modern biology.

### The emergent whole—a network of relationships

What distinguishes SGD from a simple sequence repository is the rich interconnection among data types. A researcher investigating a gene of interest encounters not a single static description but a multifaceted profile: its phenotype when deleted, its expression across conditions, the proteins it interacts with, the complexes it participates in, the pathways it contributes to, its PTMs, its localization within the cell, its abundance relative to other proteins, its regulatory inputs and outputs, and, increasingly, its relevance to human disease.

This interconnected richness emerged from decades of sustained curation, guided by the principle that biological knowledge is not a collection of isolated facts but a network of relationships. Each new data type added to SGD was integrated with existing annotations, creating a knowledge graph in which queries could traverse from sequence to structure to function to phenotype to disease.

**Figure 2:**
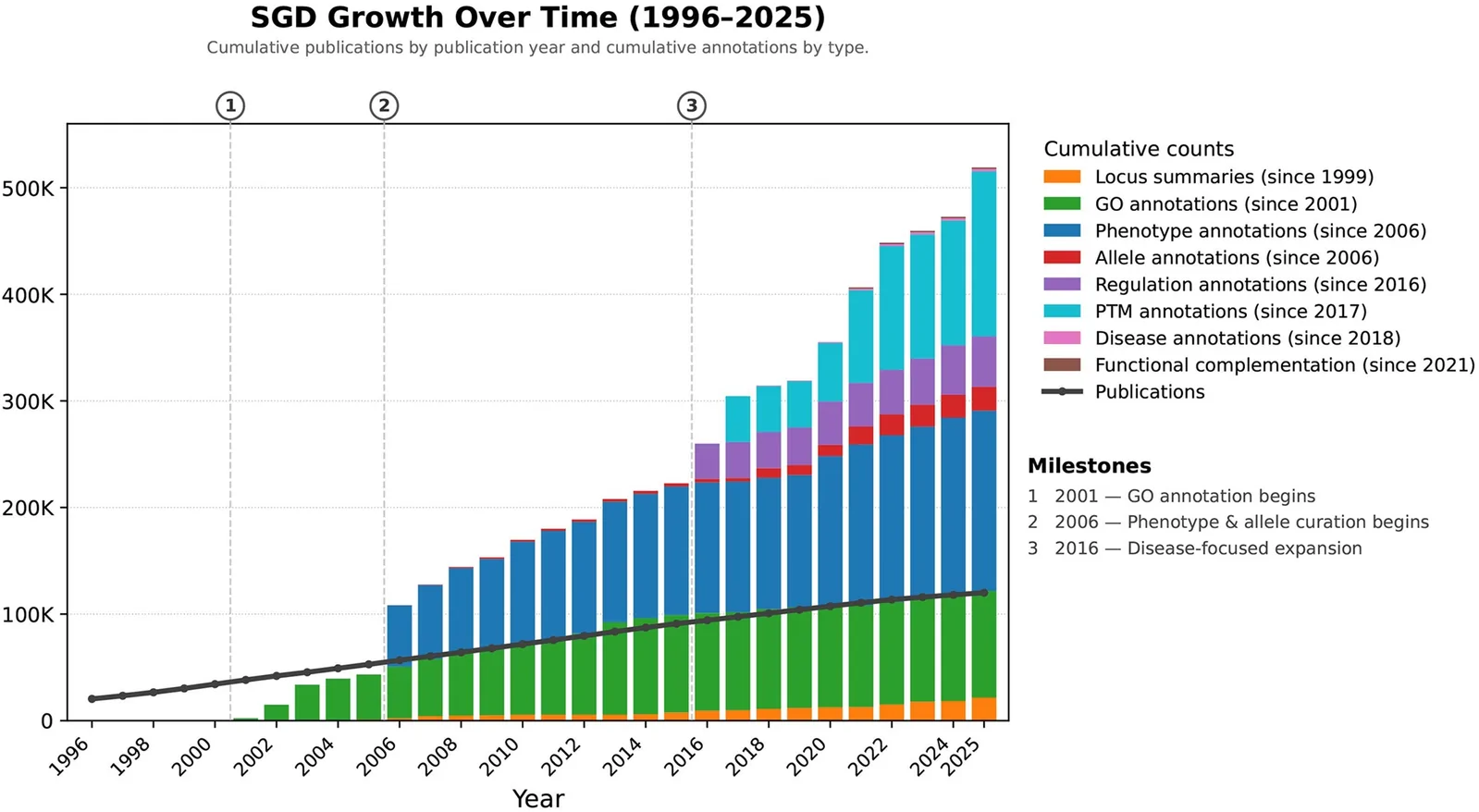
Thirty years of sustained growth in SGD literature coverage and biological annotation. The figure shows two measures of SGD’s expansion from 1996 to 2025. The black line represents cumulative papers incorporated into the database, reflecting the pace at which published yeast research has been integrated. Stacked bars show cumulative annotations across major annotation types, ordered from bottom to top by year of first curation: locus summaries (orange, since 1999), GO annotations (green, since 2002), phenotype annotations (blue, since 2006), allele annotations (red, since 2006), regulation annotations (purple, since 2016), PTM annotations (cyan, since 2017), disease annotations (pink, since 2018), and functional complementation (brown, since 2021). The progressive accumulation of annotation types, visible as new colored segments appearing over time, illustrates SGD’s transformation from a genome sequence database into a comprehensive biological knowledge resource. Three vertical dashed lines mark key milestones in SGD’s history: (1) database launch and first annotations, (2) adoption of GO, and (3) expansion into disease-focused curation. By 2025, SGD contained ~100 000 papers and over 500 000 annotations spanning diverse data types.

The community both drove and benefited from this expansion. Researchers generated the data; SGD provided the infrastructure to make it discoverable, comparable, and computationally accessible. In turn, the integrated resource enabled new research that would have been impossible without it—a virtuous cycle that has sustained the yeast research community for three decades and continues to this day (Fig. 2).

## Yeast as a model for human disease

### A pivot toward genetic medicine

In 2016, a new motivation was defined for SGD’s mission. While the goal of understanding yeast biology for its own sake remained, the project committed more fully to supporting yeast’s role in providing additional context to human disease. The biological reasoning remained the same; evolutionary conservation of protein function is conserved across a billion years of evolution. The framing, however, changed to match a clinical genomics landscape in which patients’ disease variants were increasingly being identified, and researchers needed functional context.

### Bacteria to elephants—humanized yeast

Thomas Huxley (Huxley 1869) observed that “all living forms are fundamentally of one character.” Monod and Jacob (Monod and Jacob 1961) restated the well-known axiom that “anything found to be true of *E. coli* must also be true of Elephants.” Arthur Kornberg (Kornberg 2000) noted that “mechanisms and molecules have been preserved in bacteria, fungi, plants, and animals, essentially intact.” All suggest that yeast can serve as a model for human disease. Yeast had already proven itself useful in understanding cystic fibrosis (Gnann et al. 2004), kidney disease (Lisowsky et al. 1995), Alpers disease (Qian et al. 2014), progressive external ophthalmoplegia (Stuart et al. 2006), and Parkinson’s disease (Tsika et al. 2014). New research (Kachroo et al. 2015) was showing that hundreds of yeast genes could be “humanized” by replacing them with their human orthologs and retaining function gave this argument new quantitative grounding. This “humanization” approach provided direct experimental evidence that functional conservation extended beyond sequence similarity to actual biological rescue in living cells.

### Homology pages and human disease integration

SGD developed new resources to support translational research. Homology Pages provide expert-curated evidence of functional complementation between yeast and human genes, referenced written summaries, OMIM and Disease Ontology term integration, and computationally derived ortholog tables, initially covering ∼500 yeast genes with confirmed human functional complementation (Skrzypek et al. 2018). Human disease-relevant information such as disease associations, protein domains, genetic interactions, and allele information was integrated throughout SGD’s gene pages and search tools, making translational context immediately accessible to researchers.

### Expert annotation summaries—from data to knowledge

A persistent challenge in large databases is that well-studied genes accumulate so many individual annotations that users cannot synthesize them into a coherent picture. SGD systematized the approach to this challenge: expert curators write plain language summaries across multiple dimensions of gene biology. These summaries distill complex, often contradictory literature into accessible narratives that provide context, highlight controversies, and guide interpretation.

SGD developed eight distinct summary types, each addressing a different aspect of gene function and regulation. Gene summaries provide overarching descriptions of each gene’s biological role. Function summaries synthesize what gene products do at the molecular level. Sequence summaries describe notable features of gene structure, regulatory elements, and evolutionary conservation. Phenotype summaries interpret the consequences of gene disruption or modification. Interaction summaries contextualize genetic and physical interaction data within biological pathways and complexes. Protein summaries describe protein properties, domains, modifications, and localization. Regulation summaries explain how gene expression is controlled across conditions, with particular emphasis on all DNA-binding transcription factors and transcription cofactors. Disease summaries connect yeast genes to human disease through functional complementation and mechanistic insight.

Collectively, these summaries represent a massive intellectual synthesis: expert biologists reading, evaluating, and distilling tens of thousands of primary research papers into coherent narratives. The goal is comprehensive coverage for all characterized genes, and 30 years in this goal has been largely achieved. For researchers, these summaries have transformed SGD from a data repository into a knowledge resource: a place to understand biology, not just retrieving facts.

### Allele curation and disease connections

By 2020, alleles had been elevated from annotation properties to first-class data objects. SGD had already captured >4000 *S. cerevisiae* alleles as phenotype annotation properties; now Allele pages would display sequence visualizations, DNA/protein-level data (using Sequence Ontology terms), and connections to phenotype and disease annotations (Engel et al. 2022).

The disease curation program had grown substantially: 1014 complementation relationships between yeast and human genes had been curated from 533 publications, covering 569 yeast genes and 615 human homologs. Disease annotations covered 637 yeast gene–disease associations, spanning 116 diseases and 227 human genes. This comprehensive dataset transformed SGD into a bridge between basic yeast genetics and clinical genomics, enabling researchers to leverage decades of yeast experimental work to interpret human genetic variants of uncertain significance.

### Regulatory curation at scale

SGD further expanded its curation model to encompass regulation at the transcriptional, translational, and post-translational levels. Using a data model that captured the regulator type (transcription factor, chromatin modifier, and protein modifier), the direction of regulation (positive and negative), the target gene, and the cellular context under which regulation occurs, SGD began building a comprehensive picture of the yeast regulatory network. Data from high-throughput transcription factor binding studies were incorporated alongside manually curated annotations from the classical literature (Engel et al. 2018).

## Alliance of genome resources and the maturing knowledgebase

### Twenty-six years of continuous impact

By 2020, SGD could state what would have been unimaginable in 1994: for more than a quarter of a century, the database had worked with scientists from the yeast genetics, molecular biology, and genomics communities to build a robust knowledgebase that had become essential for all aspects of yeast research. SGD was receiving nearly 1 000 000 unique user sessions per year, with page views exceeding 6.8 million annually. The Locus Summary page alone accounted for ∼50% of all page views, a testament to the enduring centrality of the gene as the organizing unit of biological knowledge.

### The alliance of genome resources

A landmark institutional development was SGD’s founding role in the Alliance of Genome Resources (Alliance of Genome Resources Consortium 2019), a consortium of major model organism databases (including MGI, FlyBase, WormBase, ZFIN, RGD, and SGD) that committed to building standardized data models, shared ontologies, and integrated cross-species search capabilities. The Alliance represented the logical next phase of the vision articulated in 1994: that model organism databases should be interoperable and mutually reinforcing, so that curated knowledge from any well-studied organism could inform research on all others.

### FAIR principles and open science

During this time SGD’s mission explicitly included FAIR principles—Findable, Accessible, Interoperable, and Reproducible—and described the database as a “modular environment” providing operational efficiencies for both internal development and external reuse. The emphasis on open access, bulk data downloads, and interoperability with other databases reflected how thoroughly the norms of data sharing had transformed the life sciences in the 30 years since SGD was founded.

## The living archive

Today, SGD’s work encompasses five major areas that collectively describe what a mature, fully realized genomic knowledge resource looks like in the mid-2020s:

- Promoting yeast-to-human disease connections through comprehensive curation of functional complementation, allele–phenotype associations, and disease ontology annotations.
- Expanding biological information on the *S. cerevisiae* genome, including regulatory relationships at transcriptional, translational, and post-translational levels, and new curated data from large-scale chromatin organization studies.
- Maintaining the authoritative *S. cerevisiae* reference genome sequence, extended to a 12-genome reference panel of widely used laboratory strains and wild isolates.
- Sustaining service to the scientific and educational communities, including gene nomenclature oversight and direct user interaction.
- Deepening collaboration with the Alliance of Genome Resources, BioGRID, and other data providers, including continued development of YeastMine as a multiorganism data warehouse.

## Continuity of founding principles

### Durable quality that evolves

SGD’s mission remains what it was in 1994: to provide the research community with comprehensive, expertly curated information about yeast genes and their functions, freely accessible to all, in service of understanding both yeast biology and its implications for human health. The specific examples have changed, with new human diseases, new experimental technologies, and new data types, but the intellectual framework has proved remarkably durable. The database that Botstein and Cherry founded in the early 1990s remains, in 2026, the authoritative knowledge resource for budding yeast genetics and a central resource for eukaryotic genomics more broadly.

## Synthesis: themes across 30 years

### The enduring primacy of expert curation

SGD has always maintained that automatic methods alone are insufficient to accurately associate biological information with genomic data. PhD-level curators who read primary literature in full, who engage personally with researchers by email and at conferences, and who develop institutional expertise over years are essential to the quality of the database. This was a contentious claim in 1994, when the field was optimistic about automated annotation. It has been validated repeatedly: poor automated annotations propagate errors, while expert-curated databases serve as quality anchors for the broader ecosystem.

### The evolution of biological annotation

SGD’s data model expanded from genetic and physical maps to a multidimensional knowledge graph encompassing GO annotations, phenotypes, protein interactions, PTMs, alleles, regulatory relationships, disease associations, and expert-written syntheses. This expansion was driven by community needs: as experimental technologies matured and researchers generated new types of data, SGD developed the infrastructure to integrate, curate, and make it accessible. The result was a transformation from a sequence repository into a biological knowledgebase where queries could traverse from sequence to structure to function to phenotype to disease.

### Productive collaboration over competition

SGD’s collaborations with other databases reflected a principle that was not always obvious in the competitive landscape of scientific research: that databases served the community better by specializing and integrating than by duplicating effort. Rather than attempt comprehensive curation of all data types in-house, SGD built partnerships with specialized databases. The collaboration with BioGRID (the Biological General Repository for Interaction Datasets) exemplified this approach: while SGD focused on comprehensive genome annotation, BioGRID specialized in curating genetic and physical interactions from the literature, using expert curators and standardized evidence codes. Rather than duplicate this effort, SGD incorporated BioGRID’s interaction data, displaying it on locus pages and linking to the detailed evidence at BioGRID. This partnership meant researchers could find interaction data through SGD while the specialized curation happened at the database best equipped to do it.

### Sharing works

The Gene Ontology Consortium operated on similar principles: shared vocabulary, distributed curation, and mutual benefit. The Alliance of Genome Resources formalized this model across six major model organism databases, with SGD as a leading member. SGD contributed not only its data but its practices such as curation standards, ontology development, data models, and software to the broader enterprise of model organism biology. These partnerships, sustained over decades, demonstrated that model organism databases could be both comprehensive and distributed, with each contributing what it did best.

## The path forward: SGD beyond 2026

As SGD enters its fourth decade, several frontiers beckon that will require both continuity of mission and innovation in approach.

### Integration of functional genomics at single-cell resolution

The yeast community is increasingly adopting single-cell RNA sequencing, spatial transcriptomics, and live-cell imaging approaches that reveal cell-to-cell heterogeneity in genetically identical populations. SGD will need to develop data models and visualization tools that capture this biological complexity, moving beyond gene-level annotations to cell-state-dependent annotations.

### Machine learning-assisted curation

While expert human curation remains irreplaceable for accuracy and biological insight, the volume of published literature now exceeds what any team can manually process in full. Natural language processing and machine learning tools that can preclassify papers, extract candidate gene–phenotype relationships, and flag potential annotation updates will augment curator capacity without replacing curator judgment. SGD has begun exploring these approaches in collaboration with Alliance partners and text-mining consortia.

### Expansion of non-S288C genetic backgrounds and wild yeast diversity

Expansion to non-S288C genetic backgrounds and wild yeast diversity. The S288C reference genome has served the community extraordinarily well, but the yeast research community increasingly works with divergent laboratory strains, clinical isolates, and wild *Saccharomyces* species. The 12-genome reference panel represents a start, and telomere-to-telomere genome assemblies now exist for multiple *S. cerevisiae* strains, providing complete, gapless sequences that reveal structural variation invisible in earlier assemblies. SGD has developed a systematic pan-genome naming scheme to manage genes and alleles across these diverse genomes (Engel et al. 2022 ). Understanding the pan-genome for any organism remains in its infancy, but the tools and approaches developed for yeast, with its smaller genome, rich genetic resources, and well-characterized diversity, can inform strategies for organisms with far larger and more complex genomes. Comprehensive pan-genome resources capturing structural variants, accessory genes, and lineage-specific adaptations will be essential for population genomics and evolutionary systems biology.

### Deeper mechanistic annotation

Moving beyond the question “*what does this gene do?*”to“*how does it do it?*” requires integration of structural biology (AlphaFold predictions and experimental structures), biochemical mechanisms (enzyme kinetics and binding constants), and systems-level models (flux balance analysis and dynamic simulations). SGD has curated protein structures and PTMs; important steps toward deeper mechanistic annotation are already underway. Recently, SGD converted metabolic pathways from YeastPathways into GO annotations via BioPAX (Biological Pathway Exchange format), enabling pathway information to be represented in a standardized, computationally accessible framework that can be integrated across databases and organisms. This approach exemplifies how mechanistic knowledge, including the specific reactions, substrates, and products that define a metabolic process, can be transformed from static pathway diagrams into queryable, interoperable data structures.

## Connecting models to data

The power of such structured, computable annotations is exemplified by recent work constructing a virtual yeast cell that integrates genomic, proteomic, metabolic, and regulatory data into executable computational models (Qian et al. 2026). Yeast’s extensive and authoritative curation to structured vocabularies and ontologies makes it uniquely positioned to drive efforts to model biology *in silico*. Once again, yeast serves as a testing ground: approaches developed for computationally modeling a well-annotated, compact eukaryotic genome can be subsequently applied to organisms with larger genomes and less complete curation. The next frontier is extending mechanistic annotation to regulatory networks, protein complexes, and dynamic simulations, making mechanistic models fully accessible and connected to primary genomic data.

### Clinical translation and variant interpretation

As human genomic medicine matures, the need for functional evidence to interpret variants of uncertain significance grows more urgent. Yeast will continue to serve as an experimental platform for testing human disease variants, particularly in conserved essential genes, and SGD’s disease curation program positions it to serve as a bridge between model organism genetics and clinical genomics databases such as ClinVar (Landrum et al. 2025) and ClinGen (Rehm et al. 2015).

### Sustainability and community governance

SGD has been continuously funded by NHGRI for 32 years, an extraordinary run that reflects both its scientific merit and the foresight of funders. But long-term sustainability requires diversification: exploring community contribution models (similar to Wikipedia or UniProt), developing premium services for commercial users while preserving free academic access, and building endowment or consortium funding structures that can outlast individual grants. The Alliance of Genome Resources represents one model; others will likely need to be explored, given the pressures on science funding.

## Educational mission expansion

SGD has always served educators and an educational role. The rise of open educational resources, interactive learning platforms, and integration with tools like Jupyter notebooks and R/Bioconductor create new opportunities, especially combined with SGD’s API that allows data retrieval. Developing teaching modules, problem sets, and datasets specifically designed for undergraduate and graduate education, and tracking their usage and impact, could formalize SGD’s role in training the next generation.

## How to choose

The challenge ahead is one of prioritization: which of these directions most effectively serves the community, advances biological understanding, and justifies continued investment? The history recounted here suggests that SGD’s greatest contributions have come when it listened carefully to its users, maintained rigorous standards, and stayed focused on its core mission: making the biology of *S. cerevisiae* understandable, accessible, and useful to the world.

## Conclusion

The *Saccharomyces* Genome Database was conceived as a solution to a specific problem: how to integrate and make useful the accumulating genomic information about a small eukaryote that was becoming the most important model organism for genetics. Thirty-two years later, it is something more: a living scientific institution with its own traditions, expertise, and community relationships, embedded in the global infrastructure of biological research.

## Everyone contributed—a global collaboration

This achievement was the work of many hands. The visionary leadership of Botstein and Cherry. The dedication of curators who built careers reading papers, talking with researchers, and maintaining the intellectual coherence of a vast database. The contributions of thousands of researchers who generated the data, corrected the errors, suggested the features, and used the resource in ways its creators never anticipated. The foresight of individuals who sustained funding across decades and multiple scientific paradigms. And the yeast research community itself, who embraced SGD not as an external service but as a shared resource: something they helped build and maintain together.

The insight at the core of SGD’s work, that the careful, expert curation of biological knowledge is itself a scientific contribution of the highest order, is now recognized as foundational to genomic medicine. It was not obvious in 1994. SGD helped make it so.
